# Forecasting Trends in Invasive Pneumococcal Disease among Elderly Adults in Quebec

**DOI:** 10.1155/2017/4347206

**Published:** 2017-01-26

**Authors:** Z. Zhou, G. Deceuninck, B. Lefebvre, P. De Wals

**Affiliations:** ^1^Quebec University Hospital Research Center, Quebec City, QC, Canada; ^2^Laboratoire de Santé Publique du Québec, Institut National de Santé Publique du Québec, Sainte-Anne-de-Bellevue, QC, Canada; ^3^Department of Social and Preventive Medicine, Laval University, Quebec City, QC, Canada

## Abstract

*Background.* In Canada, the current recommendation is to offer PPV23 to adults ≥ 65 years. PCV13 is now licensed for adults.* Methods.* Invasive pneumococcal disease (IPD) cases in adults 65–74 years of age in the Quebec notifiable diseases registry were classified into five serotype categories. Poisson regression models were fitted to monthly rates observed in 2000–2014 and predictions were made for 2015–2024, using theoretical assumptions regarding indirect effects of childhood vaccination and serotype replacement.* Results.* IPD rates caused by PCV7 serotypes decreased markedly since PCV7 introduction for children in December 2004. This trend is also underway for additional PCV13 serotypes except serotype 3. Additional PPV23 serotypes and nonvaccine serotypes have been on rise since 2004 and this is expected to continue. A small decrease in overall IPD incidence in the next decade is predicted. The proportion of PCV13 serotypes represented 33% of IPD cases in 2014 and would be 20% (95% CI: 15% to 28%) in 2024. PPV23 coverage was 53% in 2014 and is expected to be 47% (95% CI: 26% to 85%) in 2024.* Conclusion.* The potential usefulness of a combined PCV13 + PPV23 program for elderly adults would decrease over time but PCV13 would be the only option to prevent serotype 3 IPD.

## 1. Introduction

Invasive pneumococcal disease (IPD) is an important cause of morbidity and mortality in elderly adults worldwide and its burden may be reduced by pneumococcal vaccines [1]. The efficacy of the 23-valent pneumococcal polysaccharide vaccine (PPV23) to prevent IPD in immunocompetent adults has been demonstrated in clinical trials and epidemiological studies [2]. The current recommendation of the National Advisory Committee on Immunization is to offer one dose of the 23-valent pneumococcal polysaccharide vaccine (PPV23) for all individuals ≥ 65 years of age [3]. For those who have received a previous pneumococcal vaccine dose because of a medical condition that places them at highest risk of IPD, an additional PPV23 dose should be administered, as long as 5 years has passed since the previous dose. In Quebec, PPV23 has been offered to all adults ≥ 65 years of age since 2000. In provincial immunization surveys, the proportion of adults ≥ 65 years of age reporting PPV23 vaccination increased from 48% in 2006 to 57% in 2014 [4, 5].

In 2014, the 13-valent pneumococcal conjugate vaccine (PCV13) was licensed for use in adults on the basis of immunogenicity studies [6]. Results of the CAPITA randomized controlled trial in elderly adults the Netherlands demonstrated the efficacy of PCV13 to prevent IPD caused by homologous serotypes [7]. For public health authorities in Canada, the question is which vaccine or combination of vaccines to offer to elderly adults.

Pneumococcal conjugate vaccines have the potential to prevent nasopharyngeal infection and carriage in children and to reduce transmission to adults [8]. Results from a large number of studies have shown a decrease in the incidence and proportion of IPD cases caused by vaccine types in adults following the implementation of a pneumococcal conjugate vaccine programs for children [9, 10]. Another observation has been the concomitant increase in the incidence and proportion of serotypes not covered by the pneumococcal conjugate vaccines in all age groups [11]. In the province of Quebec, the universal pneumococcal conjugate vaccine program for children was launched in December 2004, and 2 + 1 schedule was recommended for low-risk children. The 7-valent CRM_197_ pneumococcal conjugate vaccine (PCV7) was first used with a catch-up vaccination for children up to 60 months of age. The licensing of new generation vaccines leads to the introduction of the 10-valent protein-D pneumococcal conjugate vaccine (PCV10) in June 2009 and the introduction of the 13-valent CRM_197_ vaccine (PCV13) in January 2011, with no catch-up in both instances.

The aim of the study was to analyze serotype-specific trends in IPD in 65–74-year-old adults in the province of Quebec in relation to pneumococcal vaccines use in children during the period 2000–2014 and to make predictions for 2015–2024, using Poisson regression models. This information is critical for an economic analysis of different immunization strategies for elderly adults.

## 2. Material and Methods

### 2.1. IPD Surveillance Data

IPD is a notifiable disease in Quebec and is defined as a clinical infection associated with the identification of* Streptococcus pneumoniae (S.p.)* by culture or nucleic acid amplification test in a normally sterile body fluid or site. IPD cases are systematically reported to regional public health authorities by hospital laboratories. Data are entered into a web-based provincial registry (MADO). In 1996, the provincial reference laboratory* (“Laboratoire de Santé Publique du Québec,” LSPQ)* initiated a laboratory surveillance program [12]. Sentinel laboratories (*n* = 21) include all tertiary care university hospitals and a sample of regional hospitals located throughout the province. These laboratories are invited to submit all their IPD isolates for bacteriological confirmation and strain characterization. Other laboratories (*n* = 68) may also transmit* S.p*. isolates on an ad hoc basis and specifically when antibiotic resistance is detected in vitro. Serotype identification is performed by the Quellung capsular swelling method. In 2014, the LSPQ invited all laboratories to transmit all IPD isolates as part of an evaluation study in ≥5 years old. All IPD cases identified by the reference laboratory are notified to regional public health authorities for registration in the MADO file. IPD cases in adults 65–74 years of age with a date of diagnosis between January 1, 2000, and December 31, 2014, were extracted from the MADO file.

*S.p.* serotypes were classified into five mutually exclusive categories: (i) PCV7 are those covered by PCV7 and including 6A (4, 6B, 9V, 14, 18C, 19F, 23F, and 6A); (ii) PCV13-7 are the additional serotypes covered by PCV13 and excluding serotypes 6A and 3 (1, 5, 7F, and 19A); (iii) ST3 is serotype 3; (iv) PPV23-13 are the serotypes covered by PPV23 but not by PCV13 (33F, 22F, 2, 8, 9N, 10A, 11A, 12F, 15B, 17F, and 20); and (v) NVT are nonvaccine serotypes. Serotype 6A was included in PCV7 serotypes as there is evidence of cross-protection induced by the 6B antigen included in PCV7. In a case-control study in the US, PCV7 effectiveness against serotype 6A IPD was 76% (95% CI: 39% to 90%) [13]. There is no evidence regarding the indirect effect of PCV13 use in children on the incidence of serotype 3 IPD in adults [14, 15]. For this reason, serotype 3 was considered as a single entity.

### 2.2. Adjustment for Incomplete Serotyping and Reporting

Forty-eight percent of adult IPD cases in the MADO file were associated with serotype identification (see Supplementary Table S1 in Supplementary Material available online at https://doi.org/10.1155/2017/4347206). IPD cases of unknown serotype were proportionally distributed into the five serotype categories for each month based on the distribution of cases of known serotype. IPD rates were computed using population census data obtained from the Quebec Statistics Institute. The overall IPD rate in the 60–79 years age group calculated from the MADO file data increased from 2000 to 2004 [16]. No such trend was seen in laboratory surveillance data [12, 17]. To take into account improvement in reporting in the period preceding the PCV program implementation in December 2004, serotype-specific IPD rates were adjusted for an annual upwards trend of 3.8% from 2000 to 2004.

### 2.3. Model Assumptions

A series of assumptions based on empirical evidence and theoretical considerations were made to predict future trends in IPD rates in adults in the context of a sequential use of three different conjugate vaccines in children:

- prior to PCV use in children, the incidence of the different IPD categories in adults was stable from year to year [12];
- there is a constant seasonal (monthly) variation in all IPD categories (Supplementary Figure S1);
- the indirect effects of PCV10 introduced in June 2009 and of PCV13 introduced in January 2011 are undistinguishable;
- PPV23 uptake in adults 65–74 years was stable during the 2000 to 2014 observation period and this will not change up to 2014 [4, 5];
- the shape of decrease in PCV7-types IPD following PCV7 introduction and of PCV13-7 IPD following PCV10 + PCV13 introduction may be approximate by a negative exponential function [14–16];
- the negative driver of the PCV7-types IPD rate in adults is the childhood PCV program computed as a function of the time elapsed since the introduction of PCV7 in December 2004, representing the indirect (herd) protection [18];
- the negative driver of the PCV13-7-types IPD incidence rates is the childhood PCV10 and PCV13 use computed as a function of the time elapsed since the introduction of PCV10 in June 2009 followed shortly thereafter by PCV13 in January 2011, representing the indirect (herd) protection [18];
- positive drivers of PPV23-13-types and NVT IPD incidences are the decreasing frequency of PCV7-types and PCV13-7-types IPD, representing replacement and assuming a constant level of invasiveness in each serotype category, plus variation in the frequency of ST3 IPD [18];
- in the base model, ST3 IPD in adults is not influenced by PCV use in children, but sensitivity analyses were performed, considering serotype 3 as a nonvaccine type (positively influenced by PCV7 and PCV13 use) or as a PCV13-7 type (negatively influenced by PCV13 use);
- the overall IPD incidence is equal to the sum of the five IPD categories for each month and year.

### 2.4. Multivariate Poisson Regression Models

All statistical analyses were performed using SAS version 9.3 software (SAS Institute, Cary, NC, USA) with a significance threshold set at 0.05. Multivariate Poisson regression models with adjustment for overdispersion were constructed using observed monthly number of IPD cases in each serotype category during the period 2000–2014.

For PCV7 and PCV13-7 IPD,

(1) Ncases=Npop·expβ0+β1·montht+β2·monthtPCV7  Introduction+β3·monthtPCV10  introduction+βseason·seasonality.

For PPV23-PCV13 and NVT IPD,

(2) Ncase=Npop·expβ0+β1·montht+β2·casePCV7+β3·casePCV13-7+β4·caseST3+βseason·seasonality.

The Quebec population 65–74 years of age was treated as an offset variable. Seasonality was adjusted using calendar months as a categorical variable. Interaction terms were tested and retained in models when they improved the goodness of fit and the Akaike information criterion [19]. Predictions were made for the period 2015–2024. Credibility intervals (95% CI) of monthly incidence estimates were computed by residual bootstrapping using 1,000 samples [20].

## 3. Results

### 3.1. Observed Trends 2000–2014

**Figure 1:**
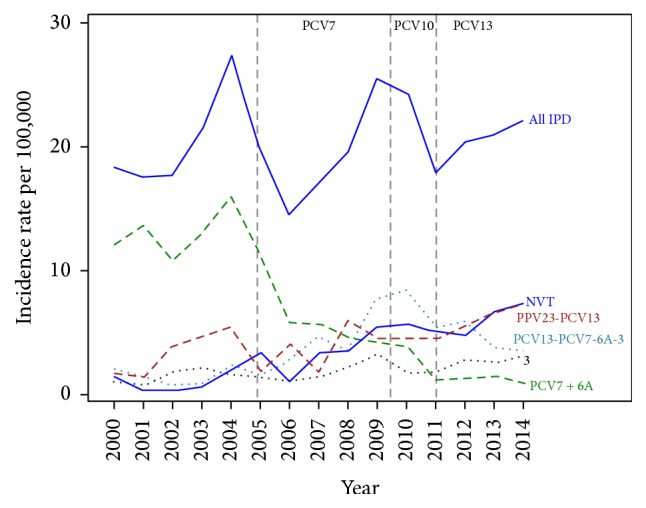
Annual incidence rate of IPD by serotype category in adults 65–74 years of age, in Quebec, 2000–2014.

During the 15-year surveillance (2000–2014), the overall IPD incidence in adults 65–74 years of age remained relatively stable (Figure 1). Incidence of PCV7 serotypes decreased markedly following PCV7 introduction in children in December 2004 and there was an increase in all other categories. Following PCV10 introduction in June 2010, followed by PCV13 in January 2011, PCV13-7 IPD incidence decreased, whereas PPV23-13 IPD and NVT IPD incidence continued to increase. Serotype 3 IPD incidence was not negatively influenced by the introduction of PCV13 in children in January 2011 and there was a slight but steady increasing trend from 2000 to 2014.

### 3.2. Predictions 2015–2024

**Figure 2:**
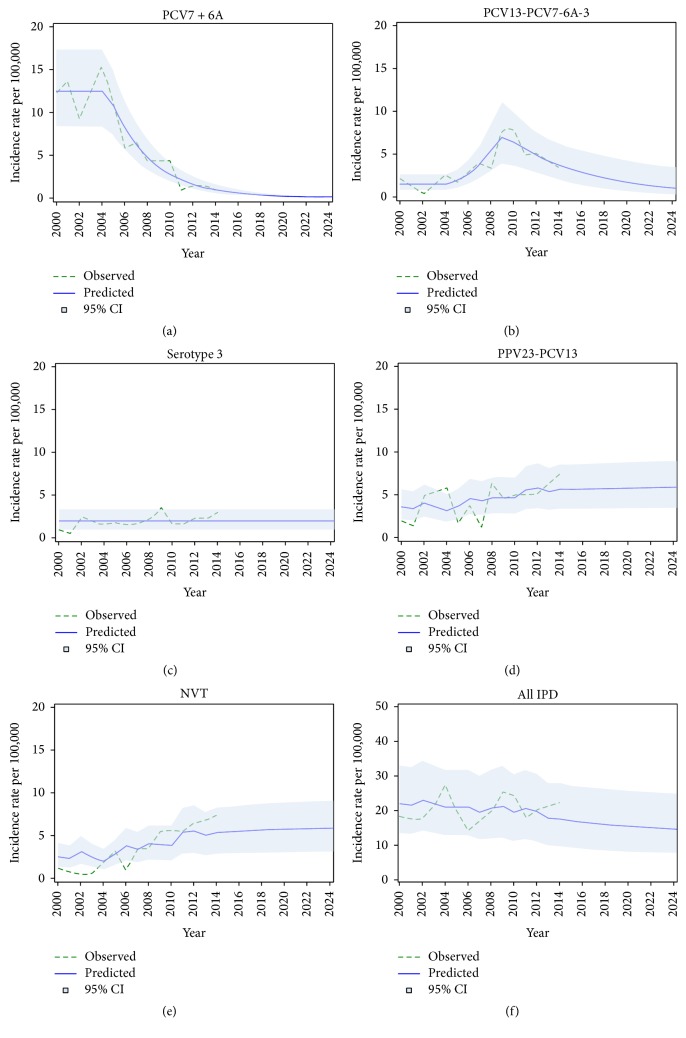
Observed (2000–2014) and predicted (2000–2024) annual incidence rate of IPD among adults 65 to 74 years of age, in Quebec (base model).

Observed (2000–2014) and predicted (2015–2024) annual serotype-specific IPD rates in the base model are shown in Figure 2 (monthly estimates in Supplementary Figure S1). The prediction is that PCV7 IPD incidence would continue to decrease in a log-linear fashion, tending to stabilize at low level. The same downward trend is predicted for PCV13-7 IPD but at a slower pace. The increase in PPV23-13 and NVT IPD would be sustained in the future with a tendency to stabilization. The overall IPD rate is predicted to decrease slightly, meaning that the herd effect would not be entirely eroded by replacement. In alternative models considering a decrease or increase in serotype 3 instead of a constant rate, results were not markedly modified (see Supplementary Figures S2 and S3).

### 3.3. Vaccine Coverage of IPD Cases

**Figure 3:**
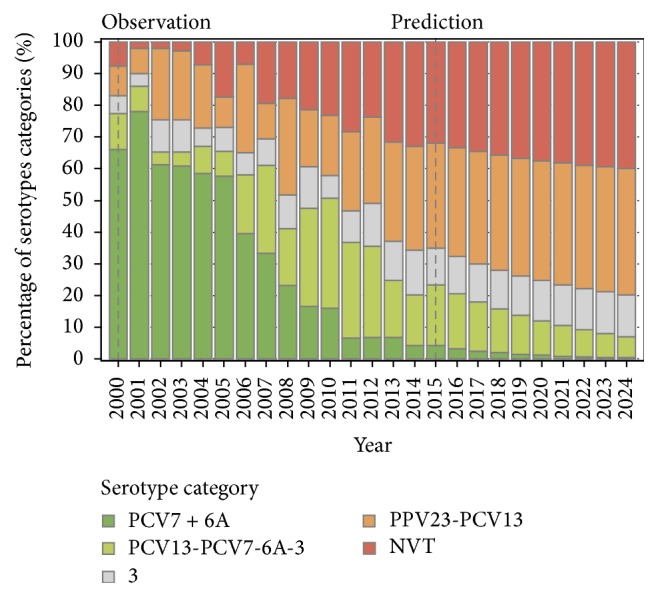
Observed (2000–2014) and predicted (2015–2024) proportions of IPD cases by serotype category among adults 65 to 74 years of age, in Quebec (base model).

The proportion of IPD covered by PCV13 and including serotype 3, decreased from 83–72% in 2000–2004 to 33% in 2014, and this downward trend is predicted to continue to reach 20% (95% CI: 15% to 28%) in 2024 (Figure 3). In sensitivity analysis including an upward or downward trend in serotype 3 IPD, the PCV13 coverage in 2024 would be, respectively, 23% [8% to 52%] or 18% [6% to 49%] (Supplementary Figures S2 to S5). The erosion in the coverage of PPV23 (excluding serotype 3) would be less pronounced, decreasing from 53% in 2014 to 47% (95% CI: 26% to 85%) in 2024 in the base model.

## 4. Discussion

Forecasting* S.p.* epidemiology should be made with great care but is a must for any economic evaluation of pneumococcal adult vaccination. Ignoring the predicted decrease in coverage of PCV13 and to a lesser extent of PPV23 following PCV13 use in children would be misleading. Results of our study suggest that the overall IPD risk in elderly adults would not be markedly reduced during the next decade, whereas PCV13 coverage would decrease from 33% to 20% (95% CI: 15% to 28%) and PPV23 coverage from 53% to 47% (95% CI: 26% to 85%). This means that postponing decisions regarding pneumococcal vaccination for adults would influence cost-effectiveness considerations very much.

A downward trend of conjugate vaccine types and stabilization at a low incidence level have also been observed and predicted elsewhere. Analysis for the UK data taking into account herd immunity from childhood programs projected a low residual conjugated vaccine types IPD in 2018-2019. They concluded that the price of PCV13 vaccine would have to be negative to obtain a cost-effective program [21]. In the US, a PCV13 + PPS23 schedule was recommended as a temporary measure based on the current epidemiologic situation and a reevaluation is expected in 2018 [22].

In our analysis, serotype 3 was considered as a special category, the relative importance of which would most likely increase over the years. Serotype 3* S.p.* has unique biochemical, phenotypic, immunologic, clinical, and epidemiologic characteristics [18, 23–25]. In Quebec, as in the US and the UK, the impact of PCV13 use in children on the frequency of serotype 3 IPD among adults was limited if any [14, 15]. In a study in the UK based on surveillance data and using the indirect cohort method, PPV23 effectiveness to prevent serotype 3 IPD in adults ≥ 65 years of age was −23% (95% CI: −85% to 19%) [26]. Estimates of PCV13 effectiveness in children (≥1 dose) were 80% (95% CI: 20% to 94%) in a matched case-control study in the US (3 + 1 schedule), 52% (95% CI: −25% to 82%) in a multicentric study in Europe (3 + 1 or 2 + 1 schedule), and 26% (95% CI: −69% to 68%) in an indirect cohort study in the UK (2 + 1 schedule) [27–29]. In the CAPITA randomized trial among adults in Netherlands, there were 4 ST3 IPD cases in the control group and only one in the PCV13 group, suggesting some level of protection [7]. Direct protection against serotype 3 IPD may be a definite advantage of PCV13 over PPV23 for adults.

Poisson regression models based on historic IPD surveillance data were applied in our study to forecast future trends. A similar approach was selected in the US and in Norway [30, 31]. Results from the ABC-CDC surveillance program showed that PCV7 introduction in 2000 was followed by a net 18% decrease in the overall IPD rate among elderly adults and after a stabilization, a further 12% decline was seen following PCV13 introduction in 2010, meaning that the herd effect outpaced replacement [15, 32]. As shown in our study, replacement was almost complete in Quebec. A slight decrease in IPD rate was observed in the 65–74 years age group from 2005 to 2014, and continuation of this trend was predicted from 2015 to 2014, suggesting that replacement is more pronounced in Quebec than in the US. We do not have a good explanation for this. More sophisticated methods have been proposed to forecast future trends in the epidemiology of IPD [33, 34]. They require, however, extensive data on* S.p.* carriage among children and adults, which are not available in Canada, and many assumptions on the competitive interactions between different serotypes in the nasopharyngeal niche and their relative invasiveness which are not robust [18].

There are strengths and limitations in our study. IPD cases were extracted from the provincial registry of notifiable disease (MADO file). IPD notification is mandatory in Quebec and systematic procedures are implemented in all microbiology laboratories to enforce this requirement. In a validation study performed in 2004–2005, 84% of adult IPD cases identified in a hospital survey were recorded in the MADO database, with no trend from year to year [35]. In the MADO file, 48% of the cases identified for the present study were of known serotype and a proportional attribution was performed for cases with unknown serotype. This could introduce a bias in the computation of serotype-specific rates if the frequency of serotyping is not uniform across serotypes and differs from year to year. Results from the validation study performed with all microbiology laboratories in 2014-2015 suggest no major bias; however, PCV13 coverage among adults ≥ 65 years was 32% (78/245) in IPD cases reported from the 22 sentinel laboratories in which serotyping was systematic and the proportion was 28% (141/503) among IPD cases from the 58 other laboratories in which serotyping was opportunistic, a statistically nonsignificant difference [36].

In our study, modeling was based on a series of assumptions which may not be entirely valid. Stability of the incidence of different serotypes categories was assumed in absence of PCV use in children although natural fluctuations in the distribution of* S.p.* serotypes spreading over decades have been described [37], as well as short-term epidemics, especially for serotypes 1 and 5 [18]. In Quebec, an outbreak caused by a virulent serotype 1 clone occurred in a few villages in the Nordic region of Nunavik in 2000–2002 [38]. However, the impact of this outbreak on the overall epidemiology of* S.p.* in Quebec was limited as the Inuit population represents approximately 0.1% of the total population of the province.

The present study is based on the epidemiology of IPD in the province of Quebec which may not be representative of all regions in the country. Laboratory surveillance data at national level are difficult to interpret as no rate is provided and the completeness of reporting has not been assessed [39]. Trends in the epidemiology of IPD in Ontario have been very similar to those observed in Quebec with a decrease in vaccine-type IPD in elderly adults following the sequential use of PCV7, PCV10, and PCV13 in children but no change in the overall IPD rate [40]. In the Calgary area, the overall IPD rate in adults decreased by 34% in adults 65–84 years of age following PCV7 introduction in 2002 [41]. More recent data would be interesting to see.

## 5. Conclusion

Although forecasting* S.p.* epidemiology should be made with great care, it is reasonable to predict further decrease in the proportion of IPD cases and rates caused by pneumococcal vaccine serotypes in Canada and replacement mostly caused by nonvaccine serotypes. Results of the present study will be used in the economic analysis of different immunization strategies currently under way.

## Supplementary Material

Supplementary materials provide the detail of serotype identification in notifiable disease registry in Québec and the results of alternative models.
