# Supplementary material for: Forecasting Trends in Invasive Pneumococcal Disease among Elderly Adults in Quebec
Source: Can J Infect Dis Med Microbiol. 2017 Jan 26;2017:4347206. doi: 10.1155/2017/4347206 (PMC5299213; doi:10.1155/2017/4347206)
Supplement: Supplementary file 1 — Supplementary materials provide the detail of serotype identification in notifiable disease registry in Québec and the results of alternative models. [file 4347206.f1.pdf]

Supplementary Material

Table S1 : Serotype identification of cases recorded in the notifiable disease registry (MADO file) in Quebec, 2000-2014

Year	IPD cases notified	IPD cases with serotype identified	% identification
**2000**	86	53	62%
**2001**	86	50	58%
**2002**	90	49	54%
**2003**	113	69	61%
**2004**	152	70	46%
**2005**	111	52	47%
**2006**	83	43	52%
**2007**	101	36	36%
**2008**	120	56	47%
**2009**	162	84	52%
**2010**	162	69	43%
**2011**	125	60	48%
**2012**	149	59	40%
**2013**	162	89	55%
**2014**	177	143	81%

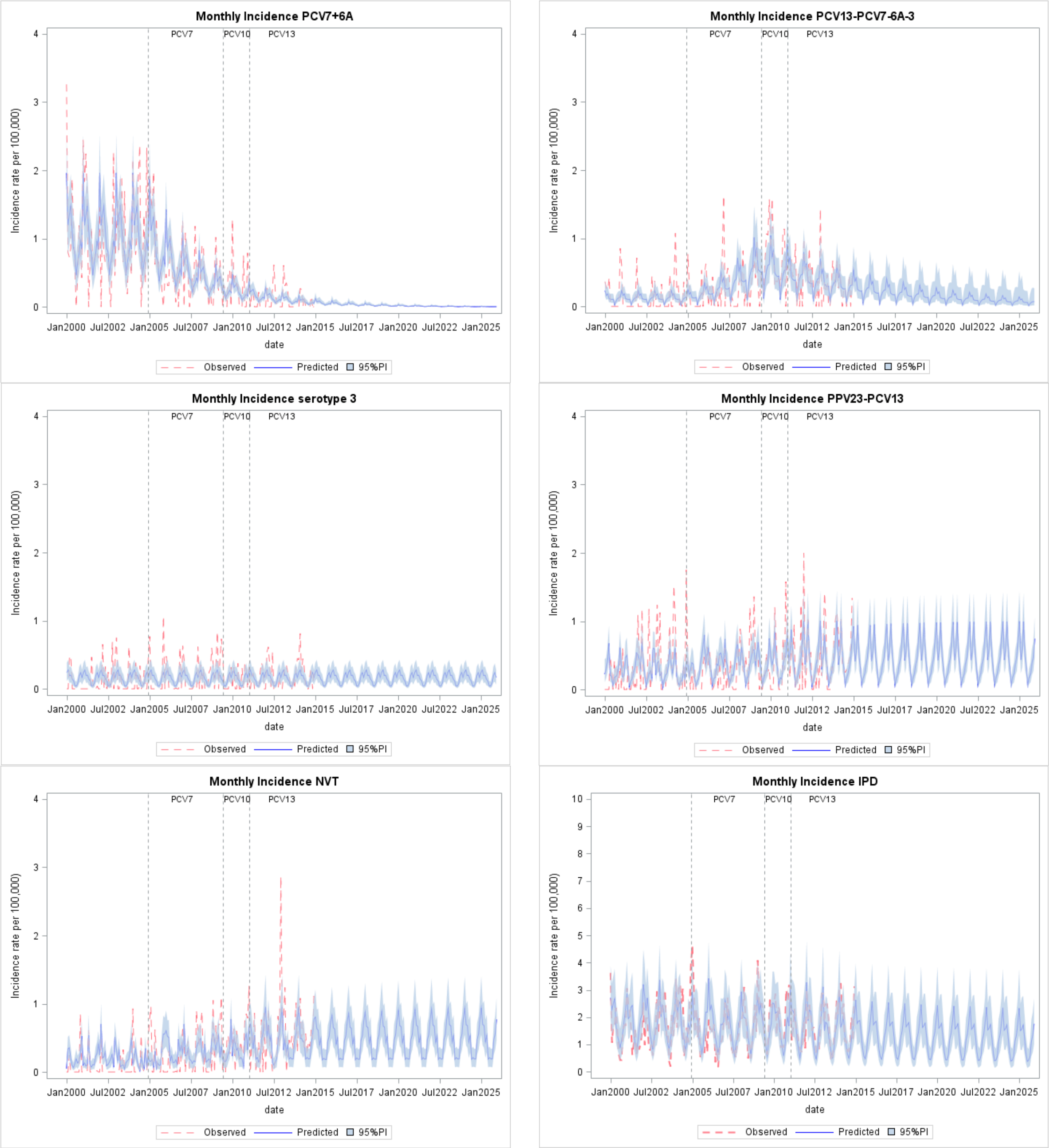

Figure S1 : Observed (2000-2014) and predicted (2000-2024) monthly IPD rate by serotype category in adults 65-74 years of age in the province of Quebec, Canada, 2000-2014 (Base model).

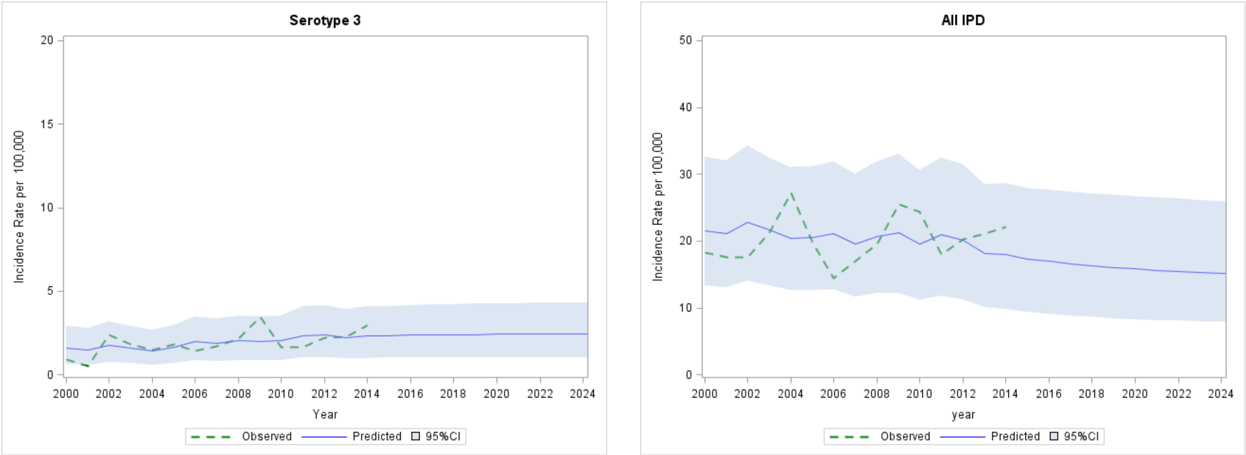

Figure S2 : Observed (2000-2014) and predicted (2000-2024) monthly IPD rate by serotype category in adults 65-74 years of age in the province of Quebec, Canada, 2000-2014 (Sensitivity analysis 1: serotype 3 considered as a NVT which is positively influenced by PCV7 and PCV13 use).

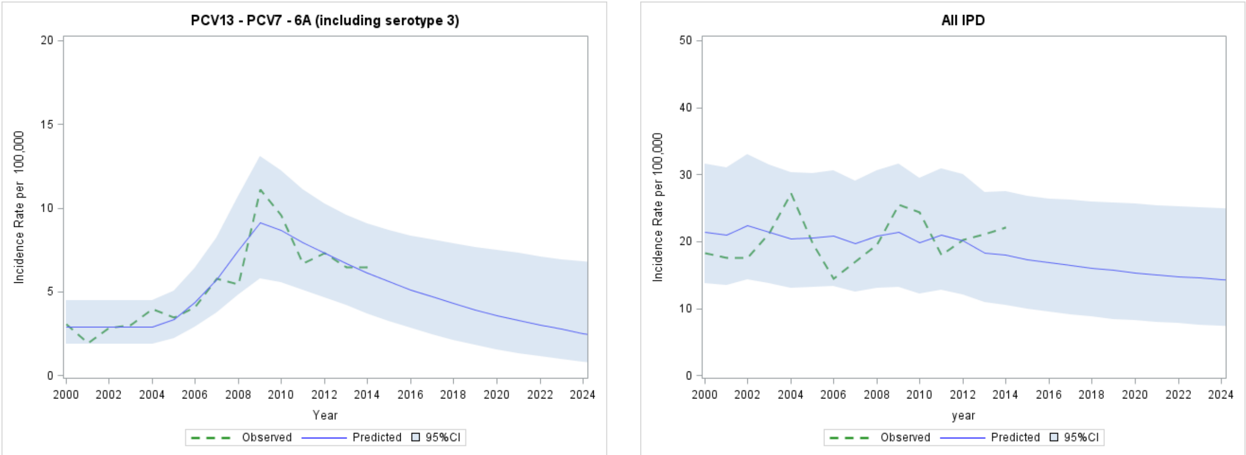

Figure S3 : Observed (2000-2014) and predicted (2000-2024) monthly IPD rate by serotype category in adults 65-74 years of age in the province of Quebec, Canada, 2000-2014 (Sensitivity analysis 2: serotype 3 considered as a PCV13-7 type which is negatively influenced by PCV13 use).

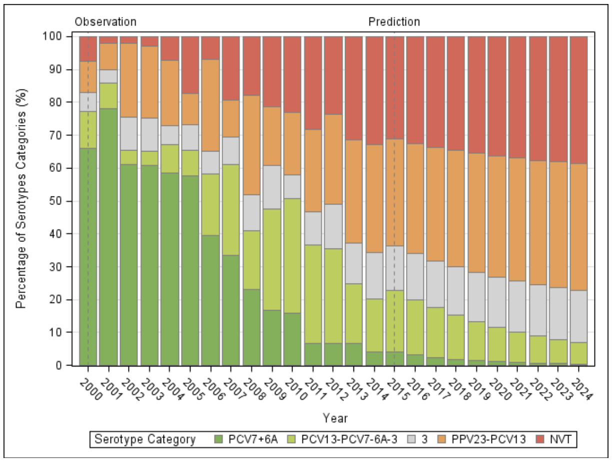

Figure S4 : Observed (2000-2014) and predicted (2015-2024) proportions of IPD cases by serotype category among adults 65 to 74 years of age, in Quebec (Sensitivity analysis 1: serotype 3 considered as a NVT which is positively influenced by PCV7 and PCV13 use).

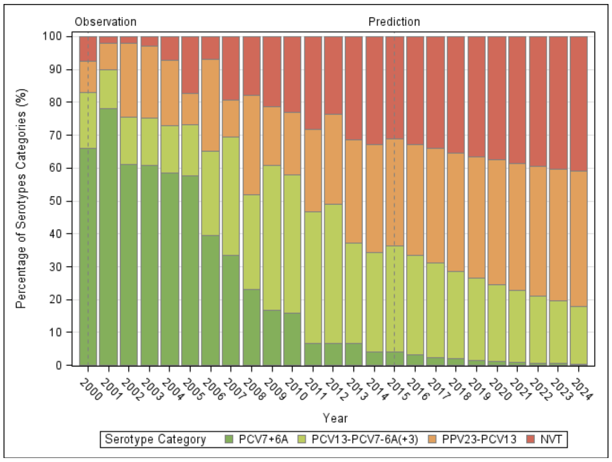

Figure S5: Observed (2000-2014) and predicted (2015-2024) proportions of IPD cases by serotype category among adults 65 to 74 years of age, in Quebec (Sensitivity analysis 2: serotype 3 considered as a PCV13-7 type which is negatively influenced by PCV13 use).
